# T-cell subset abnormalities predict progression along the Inflammatory Arthritis disease continuum: implications for management

**DOI:** 10.1038/s41598-020-60314-w

**Published:** 2020-02-28

**Authors:** Frederique Ponchel, Agata N. Burska, Laura Hunt, Hanna Gul, Thibault Rabin, Rekha Parmar, Maya H. Buch, Philip G. Conaghan, Paul Emery

**Affiliations:** 1 Leeds Institute of Rheumatic & Musculoskeletal Medicine, The University of Leeds, Leeds, UK; 2 NIHR Leeds Musculoskeletal Biomedical Research Centre, The Leeds Trust Teaching Hospital, Leeds, UK

**Keywords:** Rheumatology, Rheumatology, Rheumatoid arthritis, Rheumatoid arthritis

## Abstract

The presence of a disease continuum in inflammatory arthritis (IA) is a recognised concept, with distinct stages from at-risk stage (presence of anti citrullinated-peptide autoantibody) to diagnosis of rheumatoid arthritis (RA), including therapy-induced remission. Despite T-cell dysregulation being a key feature of RA, there are few reports of T-cell phenotyping along the IA-continuum. We investigated the disturbances of naïve, regulatory and inflammation related cell (IRC) CD4+ T-cell subsets in 705 individuals across the IA-continuum, developing a simple risk-score (summing presence/absence of a risk-associated with a subset) to predict progression from one stage to the next. In 158 at-risk individuals, the 3 subsets had individual association with progression to IA and the risk-score was highly predictive (p < 0.0001). In evolving IA patients, 219/294 developed RA; the risk-score included naïve and/or Treg and predicted progression (p < 0.0001). In 120 untreated RA patients, the risk-score for predicting treatment-induced remission using naïve T-cells had an odds ratio of 15.4 (p < 0.0001). In RA patients in treatment-induced remission, a score using naïve T-cells predicted disease flare (p < 0.0001). Evaluating the risk of progression using naïve CD4+ T-cells was predictive of progression along the whole IA-continuum. This should allow identification of individuals at high-risk of progression, permitting targeted therapy for improved outcomes.

## Introduction

Rheumatoid arthritis (RA) is a life-long progressive, autoimmune disorder that primarily affects synovial joints, affecting between 0.5–1% of adults worldwide, with 5–50/100,000 people developing RA each year. Onset is most frequent during middle age and women are affected ~3 times more. The underlying disease mechanism includes autoimmune attack on joint bone and cartilage, leading to a clinical presentation of joint pain and swelling, with consequent disability and comorbidity. Systemic inflammation contributes to an increased cardiovascular risk, which is at a magnitude comparable to diabetes mellitus^1^. Diagnosis is primarily based on signs and symptoms and the presence of autoantibodies. Current management strategies now aim to diagnose and treat disease at the earliest opportunity^2–4^.

The inflammatory arthritis (IA) disease continuum is now a well-accepted concept with distinct stages of progression (notably described as a-f phases^5^). Prior to disease, there are genetic (a) and environmental (b) risk factors that contribute to disease susceptibility, then a pre-clinical phase (of variable duration) (c), where systemic autoimmunity is manifest by the presence of autoantibodies, notably anti citrullinated peptide antibodies (ACPA), but with no clinical evidence of joint involvement. This is followed by a phase of non-specific symptoms (d) including arthralgia (joint pain) but still without evidence of clinical synovitis. For these at-risk individuals, the aim is to prevent progression to IA/RA^5^. Once clinical synovitis is detected (e, undifferentiated arthritis) individual may meet the criteria for RA depending on clinical and serological characteristics (f). However, not all individuals will progress to IA^6^ with reports suggesting up to 40–50% medium term progression from the ACPA + at-risk stage^7^. At both (e) and (f) stages, once RA is diagnosed, treatment is initiated with conventional synthetic disease modifying anti-rheumatic drugs (cs-DMARDs), usually methotrexate (MTX). The aim of treatment is attainment of clinical remission as soon as possible, to improve outcomes and minimise comorbidities^8^. This has been facilitated by improved diagnosis, use of early initiation and escalation of cs-DMARD therapy and the availability of new therapies^9^. However, diagnosing RA in sero-negative patients remains a challenge. Quantifying the risk of progression is the first step towards evidence based stratified intervention.

Although the exact pathogenesis of RA remains unclear, autoimmune processes are known to play a role, as evidenced by linkage with the Major Histocompatibility Complex^10,11^, autoantibody production^12^ and lymphocyte infiltration in synovial tissue^13,14^. These features support the hypothesis of a T-cell driven disease^15–17^, further supported by the association with many T-cells related genes^18^ and the clinical response to T-cell modulation^19^.

Over the past 10 years, our group has focused on demonstrating the value of phenotyping T-cells in the blood of RA patients. The initial discovery demonstrated the loss of naïve and regulatory CD4+ T-cells with the appearance of Inflammation Related Cells (IRC, abnormal T-cell subset expressing both naïve and memory differentiation markers)^20,21^. These data led to the consideration of whether T-cell subsets could have value as a biomarker. We established that reduced (age-normalised) frequencies of naive CD4+ T-cells in early RA at baseline were associated with failure to achieve remission with MTX treatment^22^. Furthermore, we showed that the progression towards IA from at-risk stages (ACPA + no synovitis) could be predicted using all 3 T-cell subsets^23^.

Following 2 initial reports describing the potential of T-cell subset quantification associated with 2 stages of the IA-continuum (at-risk and response to treatment)^22,23^, in this study we describe data from 705 patients recruited at distinct stages of the IA continuum, to model the potential clinical utility of scoring T-cells subset as biomarkers predictive of progression to the next stage of the continuum, with implications for targeted management. We first, modelled the clinical value of phenotyping T-cells for the prediction of progression from one stage to the next. Second, we then developed a novel scoring system for these T-cells abnormalities enabling an easy use in clinical practice. This should allow identification of individuals at high-risk of progression, for targeted management and improved outcomes.

## Results

### Quantification of T-cell subsets

**Figure 1:**
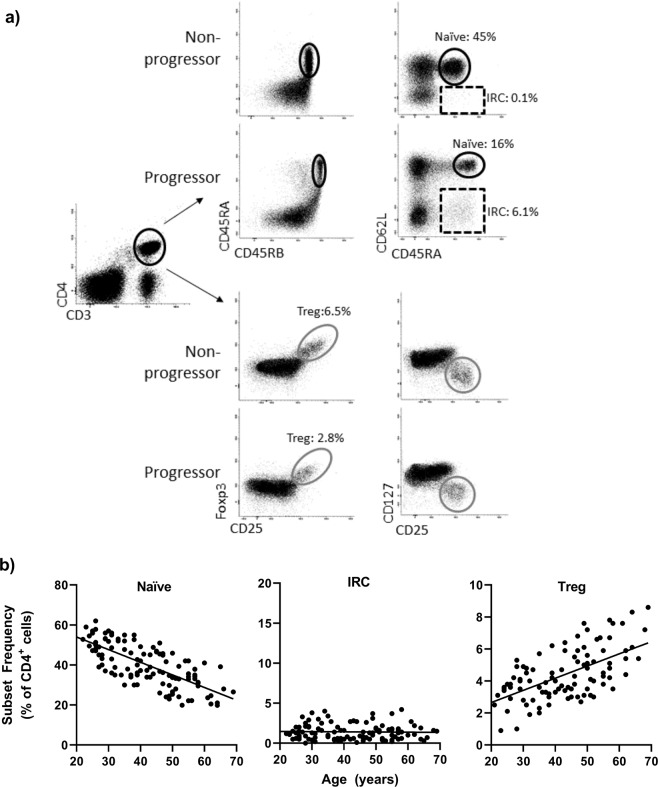
Flow cytometry analysis. (**a**) Representative flow cytometry plot for naïve (circle, CD45RBhigh/CD45RA+/CD62L+), IRC (dotted box CD45RA+/CD62L-) and Treg (grey circle FoxP3+/CD25+/CD127−) following gating on CD3+ CD4+ T-cells. Difference between health and RA are highlighted for naïve/IRC subsets. (**b**) Established age relationship in 120 healthy controls for naïve and Treg CD4+ T-cells. [expected naïve] = −0.63 ×[age] +66.6 (rho = 0.850, p < 0.0001); [expected Treg] = +0.061 ×[age] +1.83 (rho = 0.554, p = 0.001). IRC were not related to age. IRC were considered high when above the 95% CI of distribution (set at 4%).

The quantification of T-cell subsets used flow cytometry (Fig. 1, the 3 subsets were quantified as previously described based on the gates illustrated in the panel a). This technology is routinely used by hospital services and we transferred our research panels to the NHS immunology services of the Leeds Teaching Hospitals Trust. Protocols achieved comparable results (see supplementary material, Part I).

**Figure 2:**
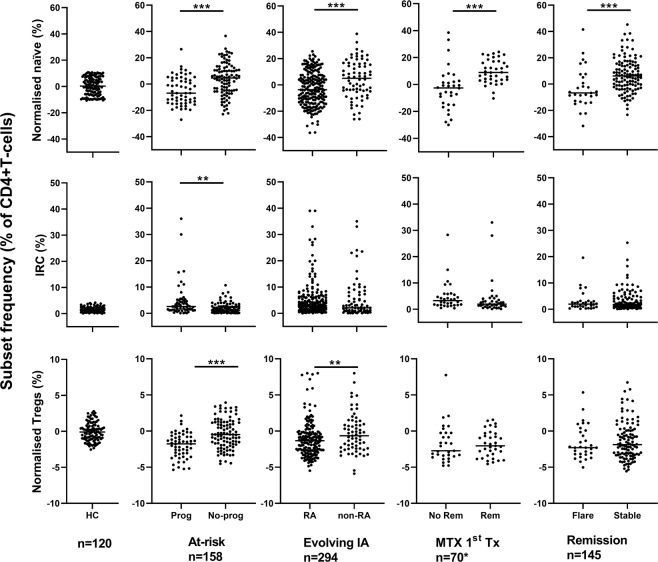
Natural history of CD4+ T-cell subset alongside the IA Continuum. T-cell subsets were quantified and data were normalised for naïve and Treg cells. Data are presented in dot plots related to the outcome at each stage of the IA continuum. Individual highly significant difference (MWU tests) are highlighted by ***(P < 0.0001) and significant difference by **(P < 0.01).

All patients were tested using the NHS services. Demographics and clinical data are summarised in Table 1 for the 4 groups. Having previously demonstrated the age relationship between naïve and Treg cells in health (depicted in Fig. 1, panel b using 120 HC), frequencies observed in patients were normalised for age as described^22,24,25^ (see also supplementary material). IRC frequencies were independent of age and dichotomised using the top of the 95% CI range in heathy control (+4%). The natural history of CD4+ T-cell subset abnormalities is described alongside the IA-Continuum, in Fig. 2.

**Table 1:** Demographics and clinical data.

	At-risk	Evolving IA	MTX 1^st^ tx	Remission
n	158	294	70	145
Age	52 (19–79)	50 (21–90)	56 (21–87)	57 (20–87)
Female (%)	117 (74%)	176 (60%)	49 (70%)	90 (62%)
Smoking*				
Current	Not collected	50 (28%)	20 (28%)	25 (19%)
Never		67 (38%)	25 (36%)	56 (43%)
Previous		59 (34%)	25 (36%)	50 (38%)
Symptom duration (m)	Na	7 (0–24)	7 (0–19)	42 (9–276)
ACPA (%)	158 (100%)	138 (55%)*	48 (68%)	90 (62%)
RF (%)	73%	121 (40%)*	42 (60%)	78 (54%)
TJC	Na	9 (0–28)	10 (0–28)	0 (0–8)
SJC	Na	5 (0–22)	5 (0–21)	0 (0–8)
CRP	Na	18 (<5–228)	10 (<5–118)	0 (<5–38)
DAS	Na	4.2 (3.2–8)	5.1 (1.8–7.5)	1.77 (0.96–2.6)

Data are described as median (range) or number (percentage). ACPA anticitrullinated peptide antibody, RF rheumatoid factor, TJC/SJC tender/swollen joint count, CRP C-reactive protein, DAS disease activity score. Na not applicable, *missing data.

We have previously reported that when patients received steroids >3 months before their baseline visit at the IA-stage of the continuum, there was no impact on the distribution of T-cell subset frequencies^25^ nor was there any significant difference in whole blood lymphocyte counts between any of the groups (data not shown).

### Individual associations with progression to the next stage of the IA-continuum

Data were first analysed using univariate methods to establish the predictive value of individual T-cells subset at each stages of the IA-continuum. In a second step, the subsets were analysed in order to classify participants for having a high risk of progression at each stage of the IA-continuum. The cut-off categorising naïve and Treg subsets for a high-risk of progression were then determined for ~80% specificity using an ROC approach.

#### ACPA+ individuals

**Figure 3:**
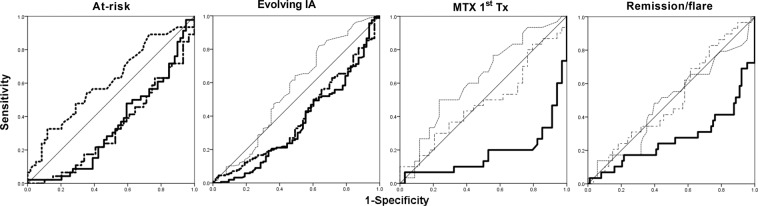
Individual ROC analysis for each T-cell subset. AUROC data are presented in Table 2. Naïve: full line, IRC: dotted line, Treg: dot-dash line. Significant AUC are indicated by bold line.

47 individuals were newly recruited since our initial study^23^. Despite not reaching high significance, we first validated previous findings, observing lower normalised naïve (p = 0.009) and Treg (p = 0.009) and higher IRC (p = 0.024) in participants progressing to IA. Combining both groups (n = 111 + 47), of which 58/158 (37%) progressed to clinical IA/RA, all 3 subsets were individually predictive (Fig. 2) with significant individual AUC (Fig. 3, P < 0.0001) and ORs above 1.95 (Table 2). A cut-off for 80% specificity was determined at −6.5% for naïve cells, −3% for Treg and +4% for IRC (Table 2).

**Table 2:** Individual subset predictive value: ROC analysis and cut-off for patients’ stratification as high-risk of progression.

		Normalised Naive	Normalised Treg	IRC	Risk-Score
At-risk participants n = 158	AUC (range)	0.313 (0.212–0.413)	0.330 (0.227–0.432)	0.313 (0.212–0.413)	3 subsets
	P	0.001	0.003	0.001	02-Mar
	Cut-off	−10%	−3%	4%	93%
	Specificity	80%	82%	80%	36.50%
	Sensitivity	41%	35%	41%	4.95
	OR	1.95	2.3	1.95	74%
	PPV	61%	64%	61%	71.50%
	NPV	63%	63%	63%	
Evolving IA n = 294	AUC (range)	0.363 (0.281–0.446) 0.001	0.389 (0.307–0.471)	NA	2 subsets
	P	−10%	0.01		Absent/Present
	Cut-off	92%	−3%		80%
	Specificity	51%	83%		69%
	Sensitivity	6.4	21%		3.38
	OR	84%	1.5		83%
	PPV	69%	80%		64%
	NPV		29.5		
MTX-induced Remission n = 120	AUC (range)	0.822 (0.710–0.933)	NA	NA	Naive subset only
	P	<0.0001			
	Cut-off	2.50%			
	Specificity	81%			
	Sensitivity	78%			
	OR	15.4			
	PPV	78%			
	NPV	81%			
Remission/ Flare n = 145	AUC (range)	0.271 (0.151–0.389) <0.0001	NA	NA	Naive subset only
	P	−2.50%			
	Cut-off	77%			
	Specificity	62%			
	Sensitivity	2.7			
	OR	43%			
	PPV	88%			
	NPV				

AUC: area under the curve (95% CI), OR: odd ratio, PPV: positive predictive value, NPV: negative predictive value, NA not applicable.

#### Evolving IA patients

Of 294 individuals studied, 219 developed RA and 75 had non-RA outcomes. Naïve and Treg but not IRC were individually predictive (Fig. 2) with significant individual AUC (p = 0.001 for naïve and p = 0.010 for Treg) and OR of 6.4 for naïve cells but only of 1.5 for Treg (Table 2). Of note, non-persistent IA resembled HC more closely that the other types of IA (statistically no difference between HC and non-persistent, while IRC tended to be higher in other IA compare to HC, p = 0.081) and all 3 subsets were statistically different to HC in RA (p < 0.05). There were however insufficient individuals in each disease category to provide a reliable comparison for UA, PsA, CTD or gout individually. On the other hand, analysing only ACPA-negative patients (RA n = 54, non-RA n = 66), the naïve (p = 0.007) and Treg (p = 0.037) subsets remained associated with the development of RA. A regression analysis is also presented in supplementary material (Part III) suggesting that a 3 variable model (naïve, DAS, age) can achieve 80% accurate prediction (all 3 contributing significantly to the model).

#### Response to 1^st^ treatment

For MTX-induced remission in early RA, our previous study^22^ had determined value only for naïve T-cells. In the new cohort recruited for this study (Fig. 2, n = 70), we fully replicated the original model, confirming the initial prediction associated with naïve T-cells (Fig. 3, AUC = 0.822, p < 0.0001 and Table 2), and the lack of added value of including Treg and IRC in the prediction model (Table 2, n = 120). For the optimisation of this outcome specific cut-off (80% specificity) using both cohorts (n = 120), it was established at +2.5% for naïve T-cells. A complete regression analysis is also presented in supplementary material (Part III, n = 120) suggesting that a 3 variable model (naïve, smoking, DAS) can achieve 88.2% accurate prediction of remission.

#### Remission

With respect to the prediction of flare (defined as increased in DAS above 3.2 (for at least 2 consecutive visits) or the need to change medication) in patients achieving clinical remission (n = 145, as defined by DAS28 < 2.6), 29 patients flared over 12months follow-up. Naïve T-cells were again the only subset with an individual predictive value with a significant AUC (Fig. 3, P < 0.0001 and Table 2). The 80% specificity cut-off was determined at −2.5% naïve cells. A regression analysis presented in supplementary material (Part III) suggests that naïve T-cell analysis can achieve 79.4% accurate prediction when considered with age and DAS (although the last 2 contribute none significantly to the model).

### Predictive value of having T-cell abnormalities

Having determined the stage specific cut-off for predicting transition to the nest stage of the IA-continuum with high specificity, we classified patients into high and low risk for each subset.

#### For ACPA+ individuals

All 3 abnormalities were individually predictive. Following optimisation of cut-off values we constructed a simple score summing the number of high-risk T-cell abnormalities (from none and up to 3, n = 150 with complete dataset). This showed an increase in the proportion of patients progressing with 2/3 abnormalities (20/27, 77%) while 0 or 1 abnormality was associated with no progression (88/123, 72%, p < 0.0001, sensitivity 36.5%, OR 4.95, Table 2, last column). No individual subset was clearly preferentially predictive of progression, although the model combining all 3 abnormalities clearly performed better than individual subset.

The proportion of patient with high-rick score (2/3) was also directly related to the proximity of symptom onset (further described in supplementary material, Part II). Furthermore, we observed worsening of the loss of naïve and Treg abnormalities with time (in 12 monthly repeated samples) resulting in a change from low-to high risk category for some patients before the onset of IA symptoms and suggesting that yearly monitoring of T-cell subset also has value (detailed in supplementary material, Part II).

#### For evolving IA patients

T-cell abnormalities were scored combining naïve and Treg (IRC not being individually predictive). The presence (1 or 2) versus absence (0) was strongly associated with RA diagnosis (p < 0.0001). Scoring for at least 1 T-cell abnormality showed prediction for progression to RA (p = 0.001, sensitivity 69%, OR of 3.38).

Diagnosing RA in ACPA + patients can now be achieved rapidly, however an early biomarker for ACPA-negative RA is still lacking. We performed the T-cell analysis in zsero-negative IA patients (54 RA/66 non-RA). Scoring naïve and Treg abnormalities still showed good prediction (p < 0.001, specificity 74%, sensitivity 62%, PPV 68% and PNV 69% and OD of 2.46).

T-cell subset quantification therefore identified which individuals presenting to an EAC clinic would progress to RA, irrespective of serology, with a score being equal to the presence of one abnormality at least, having a greater likelihood for progression to an RA diagnosis.

#### For MTX-induced remission

The score used only one T-cell subset (naïve). This new cohort fully validated our previous study^22^, allowing us to construct a model using both cohorts (n = 50 + 70). The model achieved specificity at 81.1%, sensitivity at 78.1% with an OR = 15.4 (95%CI 5.02–53.55), PPV being 78% and NPV 81%.

#### For the prediction of flare

In patient achieving clinical remission, naïve T-cells were the only subset with value. Optimising the cut-off allowed to predict flare with a sensitivity 62% and an OR = 2.7.

#### Overall IA-continuum

The predictive values (Table 2, OR) may appear modest but are significant and importantly, usable in clinical practice. During the untreated at risk stage, all 3 subsets are relevant, but after diagnosis, this changes: Treg having limited value for predicating outcome and IRC being related to levels of inflammation, become only modestly useful. The fact that naïve cells remain predictive at all stages of the IA-continuum increases their value as biomarker (i.e. a test that is predictive independent of other parameters). Combined with longitudinal data showing worsening of the naïve T-cell loss with time in at-risk patients (supplementary material) but then, a limited recovery after MTX therapy^22^, this suggest a relative stability of this phenotype over the different stages of the IA-continuum, up to the achievement of remission.

**Figure 4:**
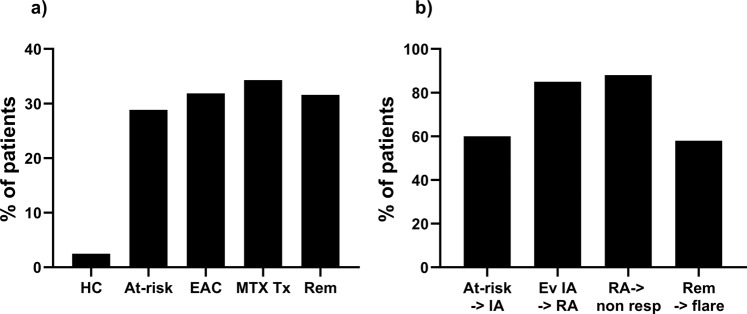
At each stage of the IA continuum. (**a)** Prevalence of participants (%) stratified as having a high-risk of progression to the next stage/outcome using naive T-cells. HC healthy control (n = 120); at-risk cohort (n = 158); EAC early arthritis clinic (n = 294); MTX tx: methotrexate treated early RA (n = 120); Rem: RA in remission group (n = 145). (**b**) Prevalence of participants (%) categorised as high-risk using naïve T-cells, who actually progressed to the next stage/outcome. At-risk -> IA: at-risk individual progressing to IA; ev IA −>RA: evolving IA patients progressing to RA; RA- > non-resp: RA patients treated with MTX, not achieving response; Rem->flare: RA patients achieving remission on sc-DMARD and flaring during follow-up.

We therefore calculated the overall prevalence of participants classified as having a high-risk of progression associated with naïve T-cells at each stage of the IA-continuum (summarised in Fig. 4a). This confirmed the relative stability of the frequency of patient with a naïve T-cell high-risk score across the different stages of the IA-continuum (23% to 33%).

The proportion of participants categorised as high-risk by naïve T-cells, who then progressed to the next stage is displayed in Fig. 4b. The lower frequency of high-risk progressors in at-risk individuals (60%) likely reflects the contribution of Treg and IRC to the overall risk while at the next 2 stages, naïve cells alone may account for most of the overall risk (85% and 88%). The low number of patient flares observed so far in the remission cohort (29/145) as well as unknown contributing factor(s) may explain the lower frequency of high-risk patients flaring at this stage (58%).

## Discussion

The current study confirms the potential value of T-cell subset quantification for predicting the progression along the IA continuum with implications for the management of IA disease.

Only a proportion of at-risk individuals develop IA, however some individuals appear to be further along the continuum than others and develop disease rapidly. Identifying such individuals at high/imminent-risk of progression is a clinical priority providing an opportunity to intervene and prevent (or at least delay) disease^5^. T-cell subset dysregulation occurs prior to the development of IA, increasing as the time to progression approaches, while remaining unchanged (or even improving slightly) in non-progressors. This opens a major opportunity for management.

For patients with evolving IA, the ability to predict those that will progress to RA should permit earlier intervention with cs-DMARDs, with well-reported benefits for long-term outcomes. Predicting RA therefore has important clinical value. The regression model suggests that using naïve T-cells offers an 80% accuracy in the prediction (with only age and DAS in the model).

In patients with new onset RA, current official guidance advocates clinical remission as a goal and suggests initial therapy with cs-DMARDs, usually MTX. Predicting response to MTX has important value in identifying patients who will do well on MTX. Conversely, identifying those unlikely to achieve this state with cs-DMARDS would avoid of the sustained inflammation which would occur with ineffective therapy, by enabling the immediate initiation of b-DMARDs (in combination with cs-DMARDs), in line with the modern treat-to-target principle^26^. A PPV of 78% for naïve T-cell status combined with a NPV of 81% therefore confirmed the potential clinical utility of naive T-cells as a biomarker for predicting MTX induced remission in early RA. In a previous study, the association between achieving remission and higher naïve T-cells was not observed in patients treated with combination MTX + anti-TNF therapy^22^. In the future, it may be appropriate to consider biologic therapy as first-line DMARD in those RA patients who, on basis of abnormal naïve T-cells, will have a low probability of achieving remission with cs-DMARDs.

Currently, for RA patients achieving clinical remission, current practice is to taper treatment aiming for drug-free remission (EULAR recommendations)^27^. Despite low sensitivity, the high specificity of abnormal naïve T-cells (confirmed by regression modelling) allows identification of patients who should not taper therapy. Further study of the depth of remission need to be performed to assess the sustainability of remission over 12 months notably when on b-DMARDs., Recent data showing that 25–30% of patients with DMARD induced remission, present with reduced naïve cells indicates the potential of this approach^25^.

Our initial hypothesis that T-cell subsets would have value as biomarker across the IA-continuum was based on a model in which the thymus in IA produces fewer naïve and Treg T-cells^20,28^. Naïve cells are also subjected to an abnormal drive for differentiation under the pressure of IL-6 and TNF resulting in the appearance of IRC^20,29^. The most specific disturbance appears to be with the naïve T-cell subset. We previously reported a similar reduction in naive cells in active compared to remitting colitis^30^, so such abnormalities may be common to immune-mediated inflammatory diseases. IRC are mostly driven by inflammation^20^ and therefore their value may be associated more closely with the monitoring of inflammation (especially when it is subclinical or when CRP is within the normal range^30^). IRCs express chemokine receptors that direct them towards inflamed tissue when disease is active^30^, but in remission, chemokine receptor expression and hyper-reactivity is decreased, so IRC re-circulate and persist in the blood. Treg-cells appear more important in the early stages of the IA-continuum. This is consistent with previous data^21^ and the recent hypothesis that the balance between Th17 and Treg cell is lost at this stage resulting in chronicity^31^. Although the identification of Th17 cells using surrogate markers (CD4+ CD161+ CCR6+ CXCR3−) is now being developed for routine quantification protocols, discrepancies exist, limiting their utility as biomarker. Alternative techniques such as those based on the epigenetic reprograming of the IL-17 gene in Th17 cells (as for FoxP3 in Treg), may bring more accurate data^32^. The combination of Th17 with naïve and Treg cells may provide an even more comprehensive representation of T-cell in the IA-continuum and increase further the predictability of T-cell subsets.

The main limitation to our work is the lack of longitudinal data due to the fact that these cohort are observational (times visits on a 3, 6 or 12 monthly basis) and progression is occurring at any time during follow-up. It is noteworthy that in the at-risk, early IA, MTX response and remission groups, a similar proportion of patients present with a high-risk (based on naïve CD4+ T-cells) (Fig. 4a). This work has successfully been transferred from research to NHS services but not yet replicated outside of Leeds and we are involved in dissemination planning. Finally, the use of the CD62L as a second marker for naïve cells (rather than other potential marker such as CCR7 or CD44) has been driven by the relevance of CD62L as a marker of exclusion from tissue expressing IL6 as much as by its role as a lymph node homing receptor characterising naïve cells^33,34^.

A study based on stratification by naïve T-cell using its predictive value for MTX-induced remission will soon start recruiting, and will provide proof of the clinical value of this biomarker. In addition to transferring to NHS-services with efficient process times and reproducibility, we also performed a second feasibility study for postal samples to allow for multicentre studies to be more easily designed (supplementary material, Part IV). The stratification of at-risk individuals developing IA/RA as well as a personalised approach to remission and tapering are now feasible, representing a significant step towards and evidence based approach to rationalising the management of RA.

## Patients, Material and Methods

### Patients

Ethical approval was obtained from National Research Ethics Committees at different phases of the IA-continuum (National Research Ethics Service, West Yorkshire Ethics Committee: REC09/H1307/98, REC10/H1307/138, NCT02433184, REC06/Q1205/169). All participants provided informed consent prior to recruitment. 120 healthy controls provided samples to establish the age-corrected healthy range of the 3 subsets^24^. Demographics and baseline data are presented in Table 1.

- At-risk individuals (n = 158): ACPA + with non-specific musculoskeletal pain but no clinical synovitis were identified as previously described^23^. Briefly, individuals were recruited when presenting with 1) a new musculoskeletal joint pain 2) absence of clinically detectable IA as diagnosed by a rheumatologist; 3) DMARD-naive. The clinical endpoint was the development of IA on clinical examination. None of the “at-risk” patient had received steroids.
- Evolving IA patients (n = 294), DMARD-naïve, were selected from our IA register between 2013–16. At 24 months, 219 patients fulfilled the 2010 EULAR diagnostic criteria and 45 patients had alternative diagnosis (ReA, AS, PSA, UA) and 30 non-persistent symptoms. Patients at baseline of the IA phase (and when receiving a 1^st^ treatment) were not on steroid although some had received an intra-muscular dose previous to being registered in the study (2 weeks to 3 months before).
- Newly classified untreated RA patients (n = 70) were used to replicate the original prediction model for MTX-induced remission and combined with 50 patients previously reported^22^, for the regression analysis. They were treated with MTX in a standardised fashion starting at 15 mg/week and escalating to 25 mg/week over 8 weeks if not achieving remission. Additional cs-DMARDs (sulfasalazine or hydroxychloroquine) were allowed if low disease activity was not achieved by 3 months. At 6 months DAS28 < 2.6 was used to define clinical remission. 37/70 (53%) achieved remission at 6 months.
- RA patients (n = 145, using the EULAR 2010 criteria) who achieved DAS28-remission (DAS28 < 2.6) and being on stable therapy for at least 6 months, having been treated only with s-DMARDs (MTX, sulfasalazine or hydroxychloroquine).

### Cell staining and flow cytometry strategies

Subset quantification was performed by the NHS-routine immunology services (in accordance to Good Laboratory Practice) for naive, IRC and Treg cell subsets. Flow cytometry was performed on fresh EDTA blood, (details of procedures and validation in supplementary material Part I). Antibody clones used are described in supplementary material Table 1S. Briefly, naïve and IRC CD4+ T-cell subsets were identified based on their expression of CD45RB-FITC, CD45RA-PE and CD62L-APC. CD4 Treg were quantified by cell surface staining for CD25-APC and CD127-PE followed by intracellular staining for FOXP3-FITC using the anti-human Foxp3 staining kit (Insight Biotechnology, Wembley, UK). Flow cytometry analysis was performed on a QUANTO cytometer (BD), using BD Biosciences FACSDIVA software. Gating was performed as previously described^22^ and showed on Fig. 1a. Subset frequencies were reported as % of CD4+ T-cells. Data analysis using CD4+ T- cell numbers for each subset (rather than % of total CD4+ T-cells) were previously discussed and did not impact conclusion or improvement of statistics. Therefore we chose not to convert % into cell number to avoid additional manipulation of the raw data.

There is an age relationship between naïve and Treg frequencies as shown in Fig. 1b. We established regression equations using 120 healthy controls for naïve and Treg CD4+ T-cells. [expected naïve] = -0.63 ×[age] +66.6 (rho = 0.850, p < 0.0001); [expected Treg] = +0.061 ×[age] +1.83 (rho = 0.554, p = 0.001). IRC were not related to age. IRC were considered high when above the 95% CI of distribution (set at 4%). For naïve and Treg, subset frequencies were then normalised^22,24^ and reported as age normalised % of CD4+ T-cells using the heathy control range: [corrected frequency] = [frequency observed in patient] - [frequency expected at that age]. The latter being calculated from the age-subset frequency correlation observed in 120 healthy control. Because this not a statistical correction but a practical variable normalisation allowing to use positive vs negative parameters, we have changed wording  (previously used in publications) to use “normalised %”: where [Normalised %] = [frequency (%) observed in patient] - [frequency (%) expected at that age] as described recently^25^.

#### Statistical analysis

Continuous variables were not normally distributed and therefore data are described using median and range (Table 2). Non-parametric tests were used throughout. Analyses were conducted using SPSS 21.1. The level of significance for P values was set at 0.05.

For univariate analysis, continuous T-cell subset measures were compared between outcome using MWU tests. No adjustment was made for multiple testing as only 3 subset variables were included at this stage. ROC analysis were then performed to establish the individual predictive value of each subset. Thresholds for the dichotomisation of subsets as high and low risk for the outcome investigated were set at ~80% specificity. Sensitivity as well as odds ratio and positive and negative predictive values (PPV/NPV) were then calculated.

A binary logistic regression model was constructed as previously described^22^ for the prediction of MTX-induced remission, for RA versus non-RA and stable remission versus flare.

Details and results at each steps are provided in supplementary material.

## Supplementary information

Supplementary material.
