# Supplementary material for: T-cell subset abnormalities predict progression along the Inflammatory Arthritis disease continuum: implications for management
Source: Sci Rep. 2020 Feb 28;10:3669. doi: 10.1038/s41598-020-60314-w (PMC7048829; doi:10.1038/s41598-020-60314-w)
Supplement: Supplementary file 1 — Supplementary material. [file 41598_2020_60314_MOESM1_ESM.docx]

**Supplementary material:**

**T-cell subset abnormalities predict progression along the Inflammatory Arthritis disease continuum:**

**implications for management**

**Frederique Ponchel^1,2^, Agata N Burska^1^, Laura Hunt^1^, Hanna Gul^1^, Thibault Rabin^1^, Rekha Parmar^1^, Maya H Buch^1,2^, Philip G Conaghan^1,2^, Paul Emery^1,2^.**

1. Leeds Institute of Rheumatic & Musculoskeletal Medicine, The University of Leeds, Leeds, UK.
2. NIHR Leeds Musculoskeletal Biomedical Research Centre, The Leeds Trust Teaching Hospital, Leeds, UK

**Part I**

**Validation of technology by NHS-routine immunology services.**

Peripheral blood was collected into EDTA (4 ml). Flow cytometry was performed as previously described[[1](#_ENREF_1)].

Staining was performed according to the NHS- routine procedures which were similar to that used previously in the research laboratory, but uses 250 ul of blood per panel whereas, all the blood were equally distributed between panels in the research setting. Over time, the original CD3 and CD4 antibody were changed to novel, brighter dies, keeping the antibody-clone unchanged.

**Table 1S: Antibody clones used for each panel.**

| Panel 1: naïve and IRC | Clone | company |
| --- | --- | --- |
| CD4-BV421 | RPA-T4 | BD |
| CD3-V500 | UCHT1 | BD |
| CD45RB-FITC | MEM-55 | Serotec |
| CD45RA-PE | F8-11-13 | Serotec |
| CD62L | 130-091-755 | Miltenyi |
| Panel 2: Treg | | |
| CD4-BV421 | RPA-T4 | BD |
| CD3-V500 | UCHT1 | BD |
| CD25-Pe-Cy7 | 2A3 | BD |
| FOXP3-AF488 | 236/E7 | BD |
| CD127- PERCP-Cy5.5 | M21 | BD |

Data were first generated in both laboratories using the same antibody panels. Gating was performed by 1 observer (FP) and directly compared with gating from the NHS-technician as observer-2 (Table S2). In a second step, staining and gating were performed on 55 samples at the NHS-routine laboratory, data were reported. Raw flow cytometry datafiles were then transferred back to the research lab and gating performed independently. Consistency was achieved for over 95% of cases between 3 observers (Table S3, average of 2 observers from NHS-services and FP). Protocols were comparable with less than 5% inter-observed variation. The slightly lower reproducibility of data for IRC is due to the low frequencies observed in most cases (median 1.0 % of CD4+T-cells).

From this work, we are confident that the panels are performing well under NHS-laboratory good practice. Discrepancy mostly related to samples with difficult pattern due to delays in transport between clinics and routine service resulting in poor quality of the sample. Work is required to produce exclusion rules for low quality samples and SOPs towards the transfer to other NHS-services.

The transfer of technology from our research settings to the routine service therefore proved viable with all data being acquired by NHS personal. Certification and adoption of the test on the NHS portfolio are under way.

**Table 2S: Reproducibility of raw flow cytometry data generated between research facility and routine services.**

|  | Naïve and IRC panel | | Treg panel | |
| --- | --- | --- | --- | --- |
| n=20 | rho | p | rho | p |
| CD3+/CD4+ | 0.870 | 0.001 | 0.911 | <0.0001 |
| Naïve | 0.968 | <0.0001 |  |  |
| IRC | 0.932 | <0.0001 |  |  |
| Treg |  |  | 0.899 | 0.001 |

**Table 3S: Reproducibility of the gating strategy between research facility and routine services.**

|  | Naïve and IRC panel | | Treg panel | |
| --- | --- | --- | --- | --- |
| n=55 | rho | p | rho | p |
| Naïve | 0.922 | <0.0001 |  |  |
| IRC | 0.799 | <0.0001 |  |  |
| Treg |  |  | 0.881 | <0.0001 |

**Part II : Details of further analysis in at-risk ACPA+ individuals.**

The proportion of ACPA+ individual who progressed to IA was 37% (58/158). Of these progressors, 40% were classified as high risk for naïve T-cells. This proportion increased with proximity to progression, being 60% in those who progressed within 6 months but ~30% in individuals progressing over longer periods.

This trend for increased risk associated with the loss of naïve T-cells was also observed longitudinally in 12-monthly repeat-testing in 26 progressors pairs, demonstrating an average yearly -3% reduction in naïve T-cell frequencies with associated change of low to high risk category for some. In contrast for 23 non-progressors pairs, the proportion of participants with high risk naïve cells was 19%, and naïve cells frequencies increased by an average of +1.7% in annual repeat sampling (n=35).

For Treg risk associated classification, 34.5% of progressors were classified as high risk (compared to 14.5% for non-progressors) and this proportion remained unchanged related to the proximity of IA onset and in 12 monthly repeat-samples.

High risk IRC were observed in 56% of progressors but this proportion increased to 65% of patients with imminent progression. In non-progressors, this proportion remained stable at 40%.

**Part III : Replication of the prediction of MTX-induced remission by naïve T-cells.**

We previously reported that T-cell phenotyping could predict remission in DMARD-naïve early RA treated with MTX [[2](#_ENREF_2)]. Reduced naïve T-cell frequency was the most predictive factor, using a cohort of 50 patients. Sensitivity/specificity of 62%/79% and Positive Predictive Value (PPV) / Negative Predictive Value (NPV) 66%/76% were reported.

For this replication study, we tested consecutive patients from an early arthritis register, representative of the general early RA population. 70 DMARD-naïve patients with early RA were treated with MTX initially. After 8 weeks, 35 patients (50%) received a second DMARD. At 6 months, 37 patients (53%) achieved remission (20/35 (57%) on MTX-monotherapy and 16/35 (48%) on combined DMARDs).

In univariate analysis, we confirmed that remission was only associated with higher naïve T-cell frequency (Table S4, 34/37 (92%), p<0.0001) and importantly in this cohort, with not-smoking (smoker vs never/previous, p<0.0001). The association between higher naïve T-cells and achieving remission was present independently of the number of DMARDs (p=0.624) although the proportion of patients with higher naïve cell frequency achieving remission on MTX-monotherapy appears higher (p=0.045, 19/20 (95%), for MTX-monotherapy and 12/17 (70%) for combination therapy). Weak associations were observed with IRC, CRP and DAS (p<0.100) and trends with TJC and symptom duration.

Logistic regression was performed to confirm that the naïve T-cell subset was associated with remission independently of potential confounders that were shown to have weak associations with remission in the above analysis (unadjusted ORs are described for reference in Table S5). A first model included 6 parameters: normalised naïve cell frequency, smoking, IRC, duration, excluding DAS but using its components CRP and TJC. The model predicted remission correctly in 79.1% of patients. It confirmed the value of normalised naïve T-cells frequency (p=0.002) and smoking (p=0.005) with CRP (p=0.016) but eliminated TJC, IRC and duration. Due to the relatively small number of patients, a second model with only 3 variables (naïve T-cells, smoking and CRP) was attempted did not increase predictability (79.1%). Replacing CRP by DAS in a third model improved predictability to 88.2% and is proposed as the best model.

Despite relatively small patient numbers in both the original study (n=50) and in this replication set (n=70), our data confirm the potential value of using naive T-cell as a biomarker for MTX induced remission in early RA. The clinical utility of measuring T-cell subsets is therefore strongly indicated by these data and suggests that measurement of T cell subsets can rationalise the use of MTX as first line therapy. One particularly interesting observation in this replication cohort was the association with not-smoking, which is in agreement with epidemiological data [[4](#_ENREF_4)]. Smoking cessation strategies could conceivably be considered in the wider multi-disciplinary early RA management pathway as an adjunct to the therapeutic regimen. Handling of smokers need future investigation as this is also a poor predictor of response to biologics [[5](#_ENREF_5), [6](#_ENREF_6)].

**Table 4S: Patients descriptive and univariate analysis**

|  |  | Remission failed  n=33 | Remission achieved  n=37 | univariate  p value |
| --- | --- | --- | --- | --- |
| Normalised Naïve* | median  (range) | -2.485  (-30.06, 39.87) | 9.19 (-10.26, 44.59) | **< 0.0001** |
| Smoking* | yes/no | 17/15 | 3/34 | **< 0.0001** |
| IRC* | median  (range) | 3.25  (0.4, 28.3) | 2.1 (0.1, 33) | 0.060 |
| CRP | median  (range) | 13.2 (0, 118) | 7.9  (0, 92) | 0.088 |
| DAS | median  (range) | 5.1 (1.8, 7.5) | 4.5 (1.8, 6.4) | 0.101 |
| Duration | median  (range) | 7.1 (0, 19.9) | 5.1 (0.8, 19.2*) | 0.156 |
| TJC | median  (range) | 11 (0, 28) | 9 (0, 28) | 0.156 |
| Age | median  (range) | 55 (21, 83) | 57 (24, 87) | 0.410 |
| RF* | pos/neg | 21/11 | 21/16 | 0.471 |
| CCP | pos/neg | 24/9 | 24/13 | 0.608 |
| Therapy | mono/combination | 15/18 | 20/17 | 0.624 |
| SJC | median  (range) | 5 (0, 20) | 5 (0, 21) | 0.701 |
| Normalised Treg** | median  (range) | -2.612  (-4.66, 7.86) | -1.982 (-4.45, 1.69) | 0.783 |
| Gender | M/F | 10/23 | 11/26 | 1.000 |

*data missing in 1 patient ** data missing in 3 patients.

The p-value was obtained from a 2-sided Fisher exact test.

**Table 5S: Logistic regression.**

|  | Unadjusted OR | Adjusted  6 Variables | Adjusted-1  3 Variables | Adjusted-2  3 Variables |
| --- | --- | --- | --- | --- |
| Smoking * | 0.0779 (0.0163,0.2732) 0.0003 | 0.0899 (0.0138,0.4280) 0.005 | 0.0918 (0.0155,0.4004) 0.003 | 0.1337 (0.0234,0.5852)  0.012 |
| Normalised Naïve* | 1.087 (1.041,1.149) 0.0008 | 1.097 (1.039,1.173) 0.002 | 1.090 (1.038,1.158) 0.002 | 1.103 (1.044,1.181) 0.002 |
| DAS | 0.6792 (0.4534,0.9782) 0.0459 |  |  | 0.5331 (0.2860,0.9020) 0.029 |
| CRP | 0.9843 (0.9640,1.0020) 0.1016 | 0.9652 (0.9339,0.9904) 0.016 | 0.9688 (0.9393,0.9924) 0.021 |  |
| TJC | 0.9597 (0.8997,1.0204) 0.1953 | 0.9258 (0.8313,1.0216) 0.134 |  |  |
| duration | 0.9438 (0.8483,1.0432) 0.2654 | 0.9514 (0.8031,1.1369) 0.566 |  |  |
| RF* (Pos v Neg) | 0.6875 (0.2543,1.8169) 0.4524 |  |  |  |
| ACPA (Pos v Neg) | 0.6923 (0.2435,1.9081) 0.4802 |  |  |  |
| SJC | 0.9692 (0.8797,1.0645) 0.5136 |  |  |  |
| age | 1.0095 (0.9782,1.0427) 0.5559 |  |  |  |
| IRC | 0.9791 (0.8969,1.0601) 0.5975 | 0.9976 (0.8915,1.1181) 0.965 |  |  |
| Normalised Treg** | 0.980 (0.780,1.229)  0.859 |  |  |  |
| Gender | 0.9731 (0.3478,2.7407) 0.9583 |  |  |  |
| **Predictive accuracy (n=68)** |  | **79.1%** | **79.1%** | **88.2%** |

*data missing in 1 patient, ** data missing in 3 patients

**Logistic regression for the early IA cohort : RA vs non-RA**

In univariate analysis, we confirmed that RA was associated with naïve and Treg subsets (Table S6, p<0.0001and p=0.030 respectively) and well as with many other clinical parameters as expected in such cohort (ACPA, RF, TJC, SJC, CRP, DAS, symptom duration, age and gender).

Logistic regression was performed to confirm that T-cell subsets were associated with RA independently of potential confounders. Unadjusted ORs are described for reference in Table S6). The EULAR 2010 criteria relies heavily autoantibodies therefore these were not included in the model. A first model included 7 parameters: naïve and Treg subsets, TJC, SJC, symptom duration, age and gender, excluding DAS. The model predicted RA correctly in 76.7% of patients. It confirmed the value of naïve T-cells (p<0.0001) but eliminated Treg, TJC, gender and duration. A second model with only demographic and clinical variables (TJC, SJC, symptom duration, age and gender) was attempted that increase predictability to 79.9%. Using DAS in a third model with naïve and age, achieved the same predictability at 80.0%. Despite the similarity in accuracy, the last model showed very low p-values for both Naïve subset and DAS while age contributed less.

**Logistic regression for the early remission cohort : flare vs stable remission**

Logistic regression was performed to confirm the value of the naive subset analysis for predicting flare. Unadjusted ORs described for reference in Table S7, suggest that no demographic or clinical data except age and DAS were also associated with flare. A single model included these 2 parameters and naïve T-cell was developed. The model predicted flare correctly in 79.4% of patients although, only naïve cells remained significant (p=0.007).

**Table 6S: Logistic regression. Early diagnosis of RA**

|  | Unadjusted OR | Adjusted  7 Variables | Adjusted-1  3 Variables | Adjusted-2  3 Variables |
| --- | --- | --- | --- | --- |
| ACPA | 5.87 (3.16, 10.92) **0<.0001** | Not used | Not used | Not used |
| RF | 3.74 (2.14, 6.53) **0<.0001** | Not used | Not used | Not used |
| Normalised Naïve | 1.040 (1.019,1.063) **0<.0001** | 1.073  (1.038, 1.109)  **0<.0001** | Not used | 1.072  (1.037, 1.109)  **0<.0001** |
| TJC | 0.913 (0.867,0.960) **0<.0001** | 0.960  (0.894, 1.024)  0.284 | 0.951  (0.896, 1.010)  0.100 | Not used |
| SJC | 0.740 (0.650,0.842) **0<.0001** | 0.810  (0.704, 0.936)  **0.004** | 0.816  (0.724, 0.923)  **0.001** | Not used |
| DAS | 0.592 (0.438,0.800) **0.001** | Not used | Not used | 0.616  (0.473, 0.802)  **<.0001** |
| duration | 0.974 (0.959,0.989) **0.001** | 0.992  (0.945, 1.021)  0.587 | 0.992  (0.968, 1.019)  0.599 | Not used |
| age | 0.975 (0.956,0.995) **0.012** | 0.969  (0.941, 0.998 )  **0.038** | 0.972  (0.951, 0.994)  **0.013** | 0.966  (0.938, 0.994)  0.017 |
| Gender | 1.590 (1.30, 1.94) **0.014** | 0.622  (0.274, 1.416)  0.258 | 0.615  (0.311, 1.019)  0.163 | Not used |
| Normalised Treg | 1.113 (1.005,1.232)  **0.039** | 1.114 (0.995,1.301)  0.072 | Not used | Not used |
| CRP | 0.988 (0.974,1.003) 0.106 | Not used | Not used | Not used |
| IRC | 1.005 (0.969,1.044) 0.790 | Not used | Not used | Not used |
| Smoking * | 1 (0.800, 1.250) 0.988 | Not used | Not used | Not used |
| **Predictive accuracy** |  | **76.7%** | **79.9%** | **80.0%** |

*missing data

**Table 7S: Logistic regression: prediction of flare in sc-DMARDs induced remission**

|  | Unadjusted OR | Adjusted  3 Variables |
| --- | --- | --- |
| Normalised Naïve | 0.933 (0.902, 0.973) **0.0001** | 0.944 (0.905, 0.984) **0.007** |
| age | 0.970 (0.941, 0.999) **0.045** | 0.990 (0.952, 1.026) 0.548 |
| DAS | 1.807 (0.838, 3.896) 0.141 | 1.776  (0.777, 4.050)  0.173 |
| TJC  SJC  CRP | Not applicable | Not applicable |
| Gender | 0.9731 (0.3478, 2.7407) 0.308 | Not used |
| duration | 1.007 (0.992, 1.022) 0.350 | Not used |
| ACPA (Pos v Neg) | 1.479 (0.638, 3.431) 0.362 | Not used |
| IRC | 0.984 (0.883, 1.096) 0.768 | Not used |
| RF (Pos v Neg) | 0.971 (0.239, 3.937) 0.782 | Not used |
| Smoking | 0.0779 (0.0163,0.2732) 0.898 | Not used |
| Normalised Treg | 1.003 (0.852, 1180  0.973 | Not used |
| **Predictive accuracy** |  | **79.4%** |

**Part IV**

**Towards clinical applicability: validation of sampling/transport procedures for centralised flow services.**

The ideal sample-handling protocol (i.e. staining fresh whole blood immediately after blood draw) is not always practical in large clinical trials. The cryopreservation of PBMCs is an attractive alternative notably for the purpose of batching samples over time, however, it has the disadvantage of depleting certain interesting cell types (such as plasmablasts and DCs), and decreasing cell yield/viability and function. Cryopreservation is also technically demanding and not all clinical sites are equipped and competent to carry it out. A case has been made for empowering individual routine services to carry out flow cytometry in large multicenter studies[[7](#_ENREF_7)]. Done correctly, this could significantly decrease the compromises associated with cryopreservation of samples. However, it requires major infrastructure commitment in terms of equipment, training and maintenance of high levels of standardization between (large) numbers of disparate laboratories to minimize variability in both acquisition and analysis of flow data. This is difficult to achieve and very costly. In many cases though, such empowerment is becoming a requirement in countries where primary samples are not usually allowed to cross borders. An alternative solution is to use some type of “preservation of samples” to allow for transport to laboratories where they will be analyses centrally minimizing the issue of standardization of the flow cytometry protocols and analysis. Several commercially available options have been proposed (i.e. Smart-tube, Trans-Fix, Cyto-Chex) to “fix” blood samples for transport however each flow-panel needs to be carefully validated before any of these tubes can be proposed as a technical solution to allow multicenter trials to use these panel.

| 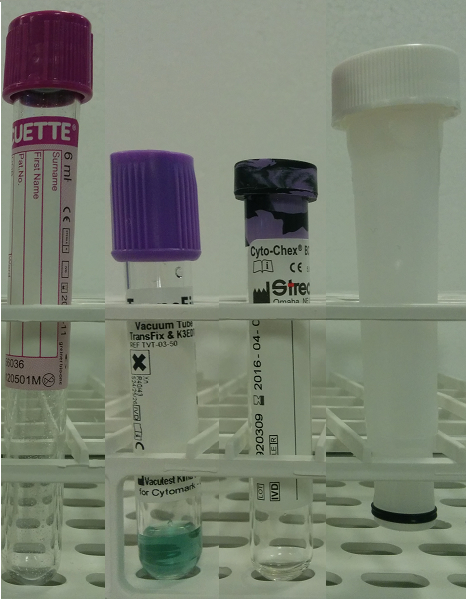 **Sm-T**  **CCx**  **TFX**  **EDTA** | Figure S3 : Fresh blood was directly collected into EDTA, TransFix (TFX), Cyto-Chech-x (CCx). An additional EDTA tube was collected and 1ml of blood was then transferred to a SmartTubes (SmT, twice), within two hours of the blood draw as recommended by manufacturers. Tubes were immediately caped and the glass ampoule (containing the fixative) inside the tube was opened by bending the tube in the middle until the ampule broke. Then SmartTubes were inverted 10 times to ensure good mixing, incubated at room temperature for 10 minutes and transferred to a -80°C freezer until analysis. SmartTubes were thawed according to manufacturer’s instructions and stained as per routine protocol. |
| --- | --- |

We designed a small study to compare data from 5 RA patient blood samples collected in these 3 types of bottles to address the performances of our main flow panels. Blood was collected and transport was mimicked by allowing the samples to rest 1 or 2 days at room temperature (manufacturer’s instructions) until staining and analysis were performed. We also added a lymphocytes subset analysis to account for possible specific subset loss over fixation/transport delays and analyzed B-cell differentiation subsets (naïve, memory, Treg) to make this study more comprehensive.

At baseline (i.e. 2 hours after blood drawing) each fixative was compared to EDTA standard blood for flow cytometry protocols. As shown in figures below, some specific markers were totally undetectable (CD45RA, CD62L, xxx) using these fixatives which is not common knowledge and not part of the limitation described by manufacturers. Lineage specificity was modestly affected although the expression of CD56 was not well conserved (not allowing to discriminate between CD56 bright and dull) and the CD3+CD56+ double positive population of NKT cells was missing in TFX and CCX tubes. Double positive cells for CD4+CD8+ were detected in TFX tube while not in the EDTA reference. The best fixative for lineage preservation appears to be SmT. For naïve and IRC CD4+T-cell subsets, 2 fixatives (TFX and CCX) totally abolished the detection of CD62L and partially that of CD45RA. The discrimination between memory cells (CD45RA-CD62L-) and other subsets, was not as good in the SmT (compared to EDTA reference) although population could still be clearly delineated. For Treg we observed relatively small changes in the shape of the population, although for some patients the FoxP3 markers was not detectable in TFX tubes while a combination of CD25/CD127 could still allow a Treg-like subset to be quantified. All B-cell markers (CD24 CD27, CD38) were detectable and although the shapes of the plots were altered, clear populations were visible in all 3 fixatives for the B-cell subsets. We then proceeded to a numerical analysis of data at baseline and over time.

For lineage subsets (Figure 2s), there was good preservation of numerical value (all 4 types of tubes) with the exception of NK/NKT cells in TFX tube in 1 patient (E, used for figure 1S). Longitudinally however, we found major discrepancies with the loss of CD19 and CD56 early at day+1 on and CD8 at day+2 after blood collection for EDTA and either one of the fixatives in 3 patients while the other 2 remained relatively stable. TFX appears to protect lineage discrimination best over time although CD56 was affected in 1 patient at baseline and CD19 lost in another at day+1/+2.

For naive and IRC CD4+T-cells, analysis was limited by the fact that CD62L and CD45RA was perturbed by the fixative while relatively intact in SmT compared to EDTA (Figure 3S). Numerically, naïve cell were quite stable over time in EDTA tubes (average variation is 10.5% of the baseline values) but IRC tended to increase by +1-1.5% at day+1/day+2 (which represents about 96% variation).

For Treg, similar results were obtained for the 4 types of tube at baseline (Figure 3S, excluding TFX). Over time, FoxP3 expression was detectable in EDTA as well as in CCX at both day+1/+2 although representing 25% variation from baseline values.

For B-cells in all 4 types of tubes, all markers being detectable the numerical evaluation showed limited variation for naïve and memory B-cell subsets although Breg were relatively less reproductively quantified (Figure 4S). Over time, variations with either increase or decrease averaged at 5% of baseline values for naïve but up to 25% for memory and Breg in all 3 types of tubes.

Altogether these results points to SmT as the best candidate choice of alternative tube for delayed/transport of samples allowing for preservation of subsets as well as sub-phonotype of both T and B-cells.


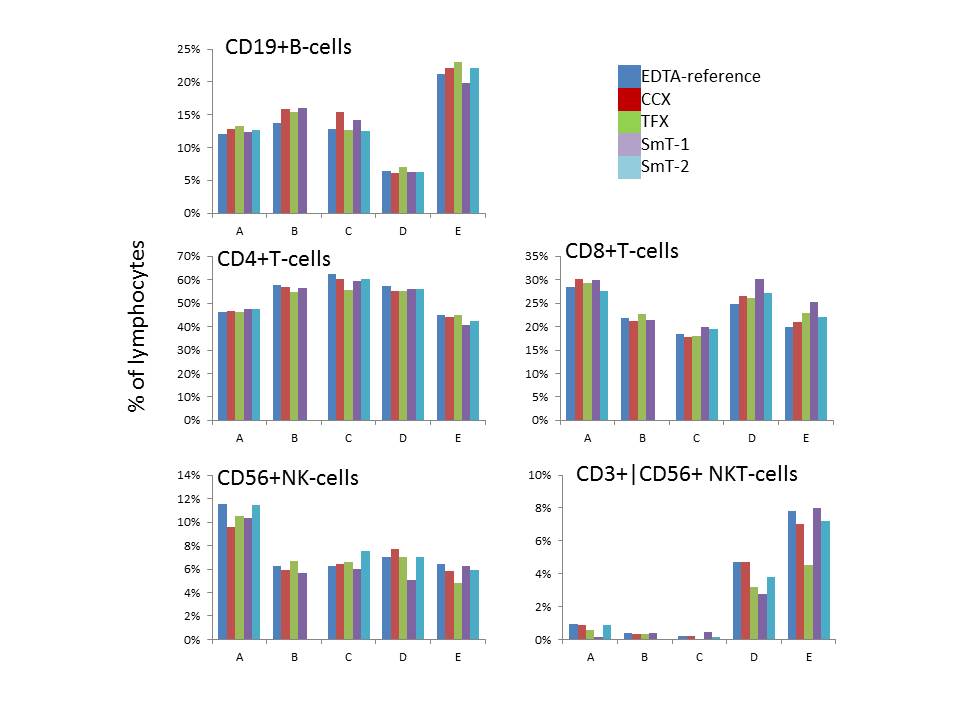


Figure 2S : Lymphocyte subsets


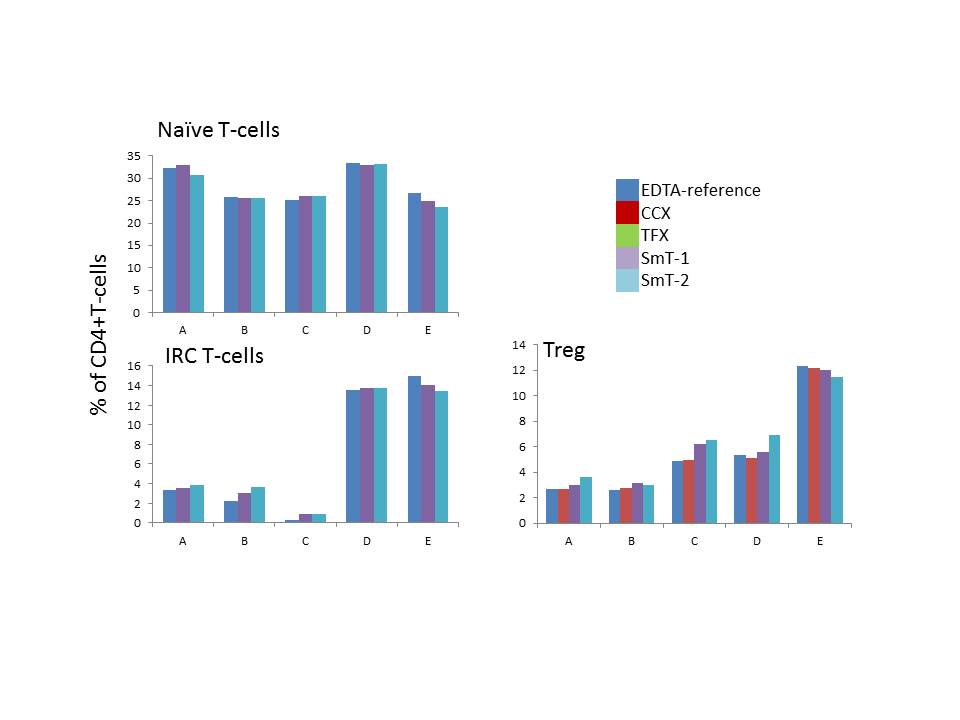


Figure 3S : T-cell/Treg panels


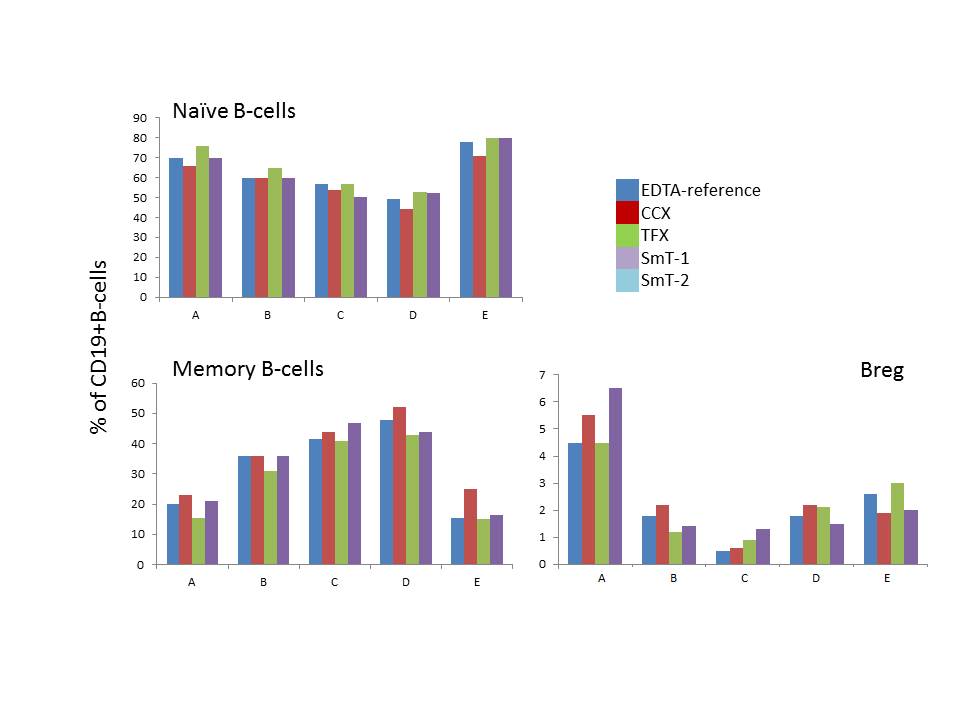


Figure 4S : B-cells panel

**References**

1 Ponchel F, Goeb V, Parmar R, et al. An immunological biomarker to predict MTX response in early RA. Annals of the Rheumatic Diseases 2013.

2 Ponchel F, Goëb V, Parmar R, et al. An immunological biomarker to predict MTX response in early RA. Annals of the rheumatic diseases 2014;73(11):2047-53.

3 Smolen JS, Aletaha D, Bijlsma JWJ, et al. Treating rheumatoid arthritis to target: recommendations of an international task force. Annals of the rheumatic diseases 2010;69(4):631-7.

4 Saevarsdottir S, Wedrén S, Seddighzadeh M, et al. Patients with early rheumatoid arthritis who smoke are less likely to respond to treatment with methotrexate and tumor necrosis factor inhibitors: observations from the Epidemiological Investigation of Rheumatoid Arthritis and the Swedish Rheumatology Register cohorts. Arthritis & Rheumatism 2011;63(1):26-36.

5 Hyrich KL, Watson KD, Silman AJ, Symmons DPM, Register BSRB. Predictors of response to anti-TNF-alpha therapy among patients with rheumatoid arthritis: results from the British Society for Rheumatology Biologics Register. Rheumatology 2006;45(12):1558-65.

6 Abhishek A, Butt S, Gadsby K, Zhang W, Deighton CM. Anti-TNF-α agents are less effective for the treatment of rheumatoid arthritis in current smokers. Journal of Clinical Rheumatology 2010;16(1):15-8.

7 Maecker HT, McCoy JP, Jr., Amos M, et al. A model for harmonizing flow cytometry in clinical trials. Nature immunology 2010;11(11):975-8.
